# Response: Neuropsychological outcome in open resection and laser interstitial thermal therapy for mesial temporal lobe epilepsy: Meta‐analysis and selective review

**DOI:** 10.1002/epi.70181

**Published:** 2026-03-09

**Authors:** Konstantina Stavrogianni, Panagiota‐Eleni Tsalouchidou

**Affiliations:** ^1^ Second Department of Neurology School of Medicine, National and Kapodistrian University of Athens, “Attikon” University Hospital Athens Greece; ^2^ Department of Neurology, Epilepsy Center Hessen, Faculty of Medicine Marburg University Marburg Germany

To the Editors,

We thank Prof. Drane and colleagues for their thoughtful commentary and for their agreement with the primary findings on language outcomes of our meta‐analysis.^1^ Below, we address their key concerns and clarify the rationale underlying our approach.

Regarding the first point, magnetic resonance‐guided laser interstitial thermal therapy (MRgLITT) is indeed a newer surgical approach than anterior temporal lobectomy (ATL) and may be influenced by variability related to surgeon experience and technique. However, it has been in clinical use for more than a decade, with outcomes reported from multiple centers. Its clinical adoption has been largely driven by comparisons with traditional approaches, which have also been evaluated in existing meta‐analyses of seizure outcomes.^2^, ^3^ Similarly, this meta‐analysis^1^ examined neurocognitive outcomes that also influence the choice between MRgLITT and ATL. Within this context, a systematic comparison between approaches is appropriate and informative, provided that differences in technique maturity are acknowledged as reflecting the current stage of MRgLITT development, and that outcomes may shift as the procedure matures over time.

Second, we agree that neurocognitive outcomes are reported heterogeneously across studies, with variation in tests, thresholds for change, and the number of measures used to define decline. To minimize this limitation, we did not pool test‐level metrics but instead analyzed the proportions of patients meeting study‐specific criteria for cognitive change. Although these criteria vary across studies, they are accepted in the literature for defining cognitive decline or improvement,^3^, ^4^ and only studies reporting objective thresholds were included. Although this approach does not eliminate heterogeneity, it avoids directly comparing disparate neuropsychological test scores. To address heterogeneity, we employed random‐effects models rather than averaging outcomes across studies. Pooled estimates were derived using logit transformation with Hartung–Knapp‐adjusted confidence intervals, a conservative approach recommended particularly when the number of studies is small (due to the fewer studies in MRgLITT) and when proportions approach boundary values (e.g., 0% or 100%).^5^, ^6^ The observed heterogeneity, already acknowledged as a limitation, also reflects real‐world clinical variability and is therefore informative for current practice.

Third, as with all study‐level meta‐analyses, adjustment for individual baseline characteristics was not possible. Individual participant data meta‐analyses could provide an opportunity to address these characteristics in the future. Regarding the studies referred to as omitted,^7^, ^8^ they were designed to develop and validate prediction models. Accordingly, they included patients with and without hippocampal resection, treating this variable as a predictor. Because hippocampal sparing substantially influences cognitive outcomes and appropriately serves the modeling objective of these publications, such cohorts could not be included in a meta‐analysis restricted to traditional surgical techniques with defined hippocampal resection. Despite their undisputed importance as foundational neuropsychological studies, these works could not provide extractable data suitable for this synthesis. Nevertheless, if eligible studies were unintentionally overlooked, we would be pleased to rerun the analysis and invite the authors to contact us.

Finally, we thank the authors for noting the concordance of the verbal memory findings with results from case‐controlled studies. While recognizing the value of controlled comparisons, all included studies met uniform inclusion criteria and underwent formal risk‐of‐bias assessment.

We are grateful for the constructive feedback and the opportunity to clarify the rationale and interpretation of our analyses.

## CONFLICT OF INTEREST STATEMENT

The authors declare no conflicts of interest. We confirm that we have read the Journal's position on issues involved in ethical publication and affirm that this report is consistent with those guidelines.
